# Four Distances between Pairs of Amino Acids Provide a Precise Description of their Interaction

**DOI:** 10.1371/journal.pcbi.1000470

**Published:** 2009-08-14

**Authors:** Mati Cohen, Vladimir Potapov, Gideon Schreiber

**Affiliations:** Department of Biological Chemistry, Weizmann Institute of Science, Rehovot, Israel; National Cancer Institute, United States of America and Tel Aviv University, Israel

## Abstract

The three-dimensional structures of proteins are stabilized by the interactions between amino acid residues. Here we report a method where four distances are calculated between any two side chains to provide an exact spatial definition of their bonds. The data were binned into a four-dimensional grid and compared to a random model, from which the preference for specific four-distances was calculated. A clear relation between the quality of the experimental data and the tightness of the distance distribution was observed, with crystal structure data providing far tighter distance distributions than NMR data. Since the four-distance data have higher information content than classical bond descriptions, we were able to identify many unique inter-residue features not found previously in proteins. For example, we found that the side chains of Arg, Glu, Val and Leu are not symmetrical in respect to the interactions of their head groups. The described method may be developed into a function, which computationally models accurately protein structures.

## Author Summary

Knowledge of high-resolution structures of proteins is an invaluable source of information for molecular biologists. Since obtaining structures experimentally is a laborious process, using computational methods to model correctly protein structure is highly beneficial. As protein structures are stabilized by the specific contacts formed by amino acid side chains, detailed understanding of inter-residue interaction is essential. Here we report a novel concept to analyze contact geometry, in which four distances are calculated between any two residues to describe their interactions in great detail. It allowed us to extract information from the Protein Data Bank, which until now was overlooked. This concept can be used to develop a computational method to accurately model protein structures.

## Introduction

Most biological activities of the living cell are directed or regulated by proteins. These diverse functions are due to proteins three-dimensional structures consequent of the physical interactions of their amino acid residues [1]. As the backbone of all amino acids is identical, side chains (and associated cofactors) dictate structure. Therefore, the knowledge of how side chains interact with each other and with the backbone will enable the computational prediction of protein structures, and the design of their shapes and functions [1].

Computational methods have been used in several research fields for the assessment and prediction of protein structures [2]–[8] such as: fold recognition, threading [9], binding [10]–[19], *de novo* design [20]–[24] and the prediction of protein stability [25]–[27]. There are two main issues to be considered while computationally modeling protein structure, one is the conformational search and the other is the scoring function. Currently rigorous functions to describe the physical interaction between molecules are computationally demanding [20],[28]. Therefore, three types of approximation are currently used: physical-based, empirical or a combination of both methods. The first is based on the fundamental analysis of forces between atoms [12], [29]–[31]. The second is a knowledge-based scheme that provides a shortcut by assuming predictable [32],[33], though theoretically questionable, potentials derived from databases of protein structures and sequences. The third method is a hybrid that combines the two schemes [34]. The majority of scoring functions are a sum of pair-wise interactions, which are assumed to be independent. Yet this approximation was proven to be inaccurate both computationally [35] and experimentally [36]. The cooperativity of the residue contacts have been modeled partially by three and four-body interaction and by modeling protein local environment with no significant advantage compared to the pair-wise methods [37]–[41].

The key concept of the knowledge-based potential (KBP) is collecting features from protein structure databases relative to random predictions [42]–[44]. Statistical potentials can be categorized on the basis of different aspects: residue-level potentials [9] *versus* atomic-level potentials [2],[29],[45],[46]. Examples of knowledge-based features used include: solvent accessibility [47], local environment [40],[41], atom contact area, sequence fragments [34], bond angle [24],[48] and distance [2], [4], [8], [14], [26], [42], [45], [49]–[51] (which is the most abundantly used). A good random model is crucial for the success of this method as being a reference state. There remains, however, a lack of consensus on how to define random models for KBP [13],[26],[28],[52]. One method is to shuffle the residues or atoms in the database and then recollect the data [46],[53]. A different scheme is the distance-scaled finite ideal gas reference state, which assumes the spatial distribution in the reference state that should be scaled as power distance (i.e. r^α^) [26]. The expected number of atom pairs in a given distance shell is proportional to that observed in the database regardless of the atom type. A few issues have been raised criticizing statistical potentials, including the argument that the topology of the proteins included in the database “remembers” the database it was derived from [54], the Boltzmann distribution assumption [32] and the reproducibility of the function using the knowledge-based method [33].

Knowledge-based distance potentials vary in the representation of the amino acid, which can be at the residue level or at the atom level. At the residue level the side chain is represented by one object; this simple model is used to reduce the computational cost, though, it was empirically shown that such simplifications reduce the accuracy of the resulting statistical potentials [2], [55]–[57]. Examples include residue centroid [45],[58] (center of mass), where a pseudo atom is calculated for each residue. Another representation for the side chain is the “volume block” for parts of the amino acid molecule [59]. In the case of atom-level representation for high-resolution models, a representative atom [60] (i.e. CA or CB) or several atom sets for each residue can be selected [61]. The collected distances are binned to a distance distribution, and the prediction power of the model is improved in accordance with the bin resolutions [5].

Here we report a new high-resolution distance method for precise description of the residue interaction geometry. We show the disadvantage of considering similar atoms as one group. Several observations regarding the reference model are described. Finally, we demonstrate that our four-distance description has predictive power on residue contact geometry.

## Methods

### Databases

High-resolution protein structures (≤2 Å) were taken from the PISCES server [62]. A 90% non-redundant dataset comprising of 6830 structures was chosen. The low-resolution protein structures (resolution 2.5 Å–3.0 Å) were also taken from the PISCES server. Non-redundant NMR protein structures with 60% identity were taken from the OCA server (1877 structures) [63]. To increase the number of distance measurements, two NMR models were chosen randomly for each structure.

### Representative atom selection

**Figure 1:**
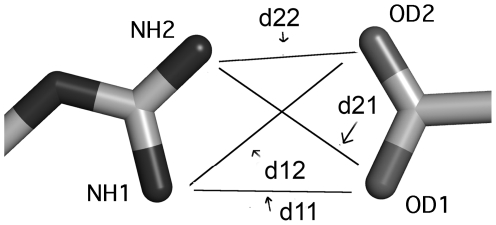
Graphical representation of the four distances for the Arg-Asp residue pair. Two representative atoms were chosen for each residue: NH1, NH2 and OD1, OD2 for Arg and Asp, respectively. Four Euclidian distances are measured: d11 for the NH1–OD1 pair; d12 for the NH1–OD2 pair; d21 for the NH2–OD1 pair; d22 for the NH2–OD2 pair.

An interaction between two residues is defined in terms of four distances between two pairs of atoms (Figure 1). The following notation “R1_R2_A1_A2_B1_B2” is used throughout this study, where R1 and R2 are the interacting residues, A1 and A2 is the pair of atoms in residue R1, B1 and B2 is the pair of atoms in residue R2. For example, Lys_Val_CB_CD_CG1_CG2 means that for the interacting residue pair Lys and Val the representative atoms of Lys are CB and CD and the representative atoms of Val are CG1 and CG2. To choose the pair of atoms for each residue pair, all the possible combinations were enumerated. The four Euclidean distances were collected for all of them and a four-distance set was kept only if one of the distances was <5 Å. This cutoff enabled us to discard the huge amount of data comprising distanced non-interacting pairs. A representative four-atom set was chosen for each residue pair. The criterion for choosing this representation was to maximize the chances that at least one interactive distance of less than 5 Å will be represented for each amino acid pair (Table S1). This allows the possibility that the same amino acid is in contact with different partners via different atom pairs. For example, the Lys–Val contact was defined as Lys_Val_CB_CD_CG1_CG2 while the contact of Lys with Asp was defined as, Lys_Asp_CD_CE_OD1_OD2. Side chain backbone contacts were grouped regardless of the identity of the backbone amino acid. The representative backbone atoms are the alpha carbon and the carboxyl oxygen. For example, for Arg_Any_CG_NH2_CA_O side chain of Arg can be in contact with backbone atoms from any residue.

### Random model

The random model was generated from the high-resolution dataset (see above). Each residue conformation was replaced randomly by a rotamer from the rotamer library [64], keeping the same amino acid identity. No considerations for rotamer probabilities or conformational clashes were taken. The distances were collected as for the real data. However, to reduce the noise level of the random data, a 25-fold excess of random to real data was used.

### Histogram setup

The four distance data were arranged in a 4-D histogram. Each contact pair was binned at a resolution of 0.5 Å, from 0 to 10 Å forming histograms of 21^4^ bins (i.e. 194481). The probability (frequency normalized to one) for each four-distance combination was the number of measurements in a selected bin divided by total number of measurements.

### The flying rotamer method

To test the suitability of the derived data for modeling residue-residue interactions a simulation was performed. Two residues with arbitrary side chain conformations were built. Then, these residues were randomly placed relative to each other 10^6^ times. Four distances between atoms defining the residue-residue interaction were calculated for each random placement and a score was assigned in accordance with Equation 2. The orientation of two residues, for which the best score was attained was identified and examined.

## Results

**Figure 2:**
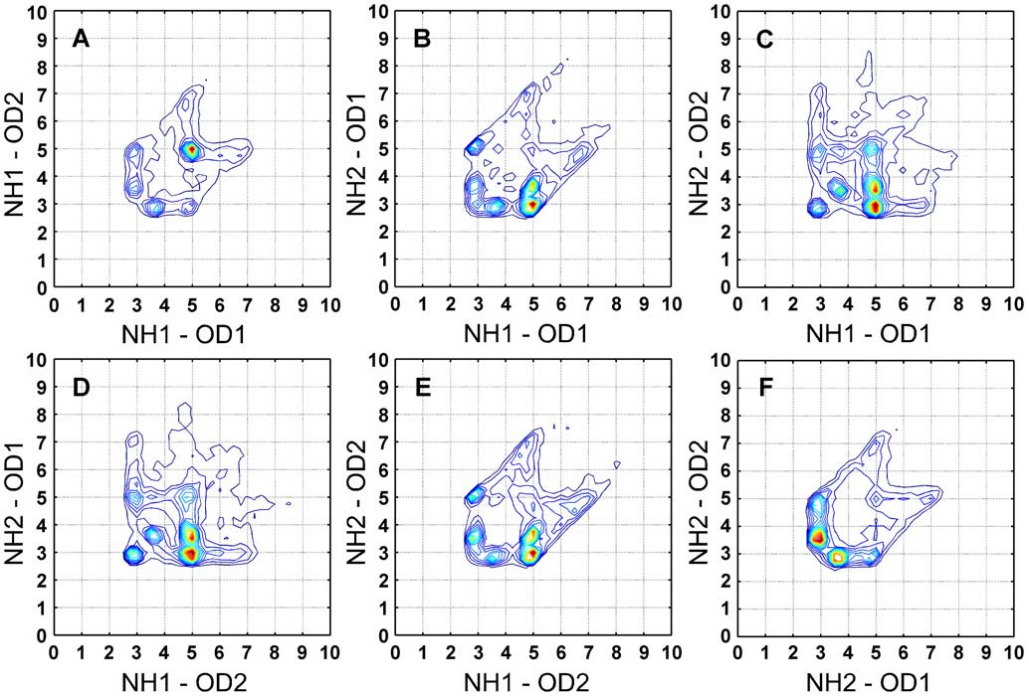
Six projections of the four distances connecting Arg and Asp. The four distances are: NH1–OD1 (d11), NH1–OD2 (d12), NH2–OD1 (d21), and NH2–OD2 (d22). Six projection are needed to plot the four dimensional data. Only cases with correct IUPAC atom name assignment protocol were considered.

Multiple distances between two bodies can describe the exact spatial relation between them. For amino acids, a further complexity arises from the flexibility of their construction, which results in many possible rotamers per amino acid. Theoretically, this would require multiple distances per amino acid pair to achieve a complete description of their spatial relation. However, due to the high dimensionality of the problem this would result in bins scarcely populated when using the known protein structures in the PDB. Therefore, we described the relation between any two amino acids in terms of four distances. These four distances were calculated between two atoms from each residue (Figure 1). For example, the interaction between Arg and Asp was defined as follows: Arg_Asp_NH1_NH2_OD1_OD2. This means that for the Arg–Asp interaction pair the representative atoms are NH1, NH2 for Arg and OD1, OD2 for Asp, namely atom 1 and 2 for each residue (for more details see Methods). The four distances describing the contact between the two residues are therefore: NH1–OD1 (d11), NH1–OD2 (d12), NH2–OD1 (d21), and NH2–OD2 (d22) (Figure 1). Assuming the four distances are independent, this method provides a four dimensional description of each interaction. The four-dimensional data can be viewed using six two-dimensional projections as shown in Figure 2 for the Arg–Asp pair. The 4-D data were collected and binned for the 19 amino acids (excluding Gly) resulting in 190 pairwise combinations. In addition, we collected 18 interactions between side chains and backbone atoms (Table S1). The 4-D data were collected from a set of 6830 protein structures (sequence identity <90%) [62]. In the case of multiple conformations of the same amino acid, we considered only the first conformation for data collection. We found that less than 1.5% of analyzed residues in our dataset have alternative conformations. This experimental dataset provided a sufficiently large source of information required to construct a database of the four-distances, binning the data with intervals of 0.5 Å.

### Asymmetry of amino acid

The 4-D description of residue-residue interactions is a more restricted form of the single distance potential extensively used. This higher order description allowed us to challenge common beliefs of the symmetric nature of some of the amino acids. For example, the Arg nitrogen atoms NH1 and NH2 were usually considered to be exchangeable with one another [55]. The notation of atoms for Arg in X-ray structures of proteins is as shown in Figure 1: in the guanidinium plane NH2 is trans to CD [65]. In Figure 2 we present the distances between the Arg–Asp atom pair as collected from the PDB. Comparing the two distance peaks of NH1–OD1 (d11) and NH2–OD1 (d21) (Figure 2B) shows a non-equal distribution of the two populations: d21 at 3 Å and d11 at 5 Å is much more populated than d21 at 5 Å and d11 at 3 Å. This observation shows that the guanidinium group in the Arg residue is not symmetric [55], which is a result of the notation of Arg in the PDB: in this conformation NE can serve as a hydrogen bond donor (compare Figure 2A to 2B). In the conformation where d21 is 5 Å and d11 is 3 Å the CD atom sterically interferes in forming the second hydrogen bond of an incoming residue with NE. Figure 2E gives the interaction distances for the atom pairs NH1–OD2 and NH2–OD2, showing that OD1 and OD2 are symmetric while NH1 and NH2 are not. It is interesting to note that it is rare to find OD1 and OD2 being located at an equal distance from either NH1 or NH2 (Figure 2B, 2E and 2F). In fact, one distance is 3 Å while the other is 3.6 Å. This means that at a given Asp–Arg interaction only one of the OD atoms forms a hydrogen bond with the NH group of Arg, while the second distance is beyond the hydrogen bond threshold and may be of an electrostatic nature. To the contrary, the distances of both NH1 and NH2 with either OD1 or OD2 peak at 3 Å (Figure 2C and 2D).

**Figure 3:**
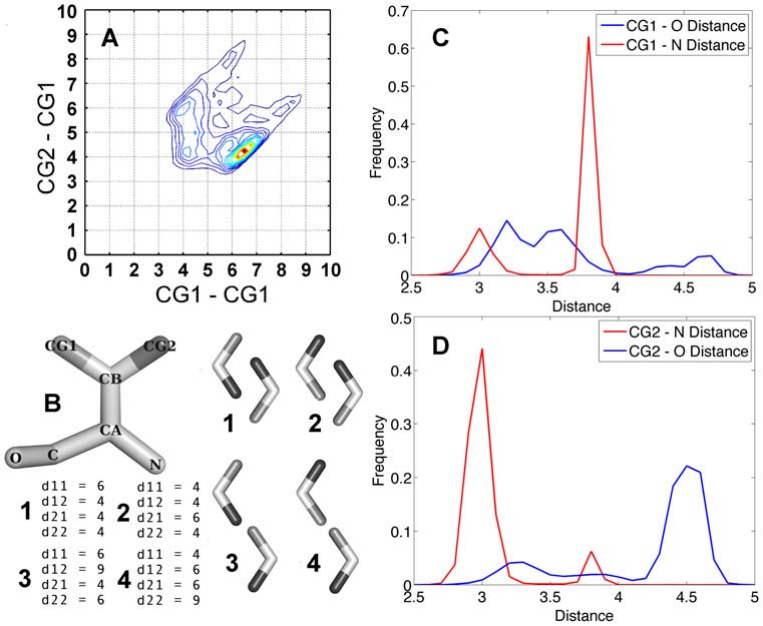
Val asymmetry. Only cases with correct IUPAC atom name assignment protocol were considered. (A) The CG2–CG1 distance (d21) has a higher occupancy at 4 Å than the CG1–CG1 distance (d11). (B) Val atom notation in the PDB. The CB atom is a prochiral atom, putting the CB hydrogen at the back, and starting at clockwise direction from the CA atom the following atoms are always CG1 then CG2. (B) The V shape objects represent the covalent bond between CG1 CG2 and CB in the Val side chain. The possible Val conformation with CG1–CG1 (d11) and CG2–CG1 (d21) distances of either at 4 Å or at 6 Å (C,D). Two histograms depicting the distance between CG1 (C) and CG2 (D) from the backbone nitrogen and oxygen. (C) CG1 atom is generally closer to the backbone oxygen. (D) CG2 atom is generally closer to backbone nitrogen.

Another striking example of an amino acid, which is considered as symmetric, is Val. Defining the atom notation as an optical enantiomer with CB acting as a pseudo chiral center and the hydrogen pointing away from the viewer, then going clockwise from CA the two methyl groups are always CG1 and next CG2 [65]. In Figure 3A one can see that the shorter d21 distance (CG2–CG1) of 4 Å is preferred over the d11 distance (CG1–CG1) of 6 Å. Figure 3B (panels 1 to 4) depicts the possible orientations where d11 and d21 are 4 Å or 6 Å. The observation that the B1, B3 conformations are preferred over the B2, B4 conformations require an explanation. Figures 3C and 3D suggest that CG1 is generally closer to the backbone oxygen (negative partial charge) while CG2 is closer to the backbone nitrogen (positive partial charge). In the B1 conformation there are three short bonding distances, two with opposite partial charges and one with a similar charge. Conversely, in B2 there are two distances with a similar partial charge, and one with an opposite partial charges. For the same reason, B3 is preferred over B4. The definition we propose in the 4 dimensional matrices exploits this asymmetry because the two atoms are treated separately. The two examples brought here demonstrate how defining interactions as a 4-D matrix provides high-resolution structural insight.

**Figure 4:**
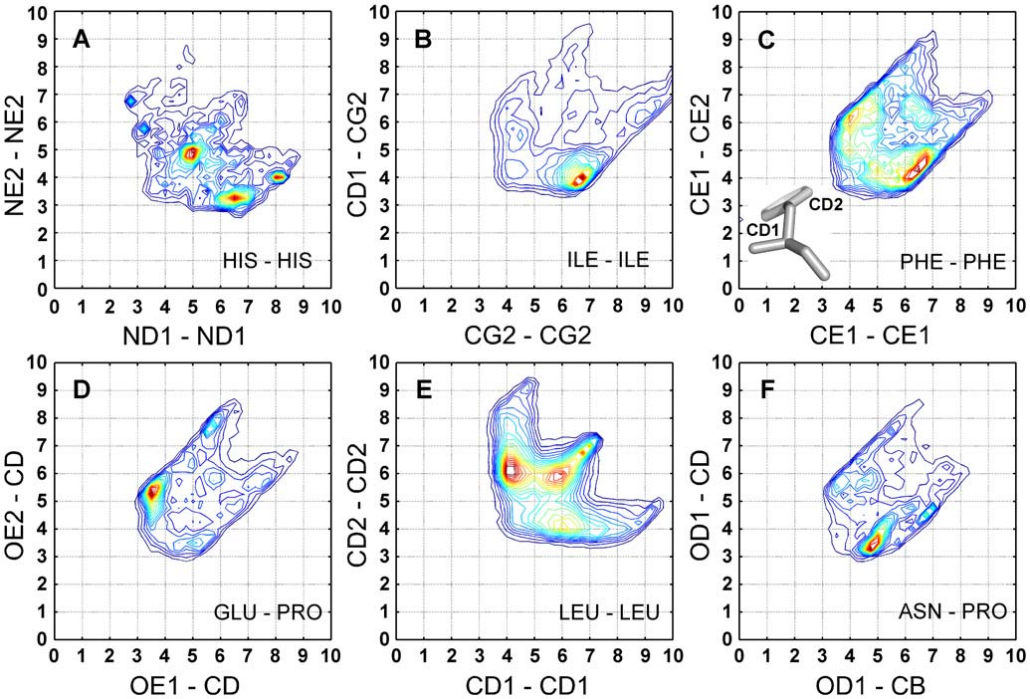
Asymmetry in 4-D distribution of His, Ile, Phe, Glu, Leu, and Pro residues. Only cases with correct IUPAC atom name assignment protocol were considered. (A) The NE2–NE2 distance of the His-His pair has a higher occupancy around 3.5 Å compared to the ND1–ND1 distance. (B) The CD1–CG2 distance of the Ile-Ile pair has a higher occupancy around 4 Å compared to the CG2–CG2 distance. (C) The CE1–CE2 distance of the Phe-Phe pair has a higher occupancy at around 4 Å compared to the CE1–CE1 distance. The CD atom that makes the smaller torsion angle is named CD1 (see inset). (D) The OE1–CD distance between Glu and Pro has a higher occupancy at the 3–4 Å interval compared to the OE2–CD distance. (E) The CD1–CD1 distance of Leu-Leu has a higher occupancy at around 4 Å compared to the CD2–CD2 distance. (F) The CD1–CD1 distance of the Pro-Pro pair has a higher occupancy at around 4 Å compared to the CD2–CD2 distance.

Additional examples of the asymmetry of the same atom types behaving differently in different contexts include His, Ile, Phe, Glu, Leu and Pro (Figure 4, panels A–F). We have noticed different distance distributions for the two nitrogen atoms of His. The NE2–NE2 distance has a higher occupancy at 3.5 Å (which is a good hydrogen bond distance) compared to ND1–ND1 (Figure 4A). For Ile, the CD1–CG2 distance is more occupied at 4 Å compared to the CG2–CG2 distance (Figure 4B). The origin for this discrepancy might be the better availability of the CD1 and NE1 atoms over CG and CD1 for the Ile and His residues respectively, as being farther away from the backbone on the residue side chain. In the Phe 4-D distribution, shorter d12 distance (CE1–CE2) of 4 Å is preferred over the d11 distance (CE1–CE1) of 6 Å (Figure 4C). This example holds also for Tyr. In the histogram of the Glu–Pro contacts it seems that OE1 is preferred over OE2 bonding to the Pro CD atom. Phe and Glu are similar residues in the sense of having a symmetrical side chain, the atom names were defined by the second chi angle [65]. As can be seen in the inset of Figure 4C, the atom that makes the smaller chi angle is assigned number 1 [65] (in the figure it is CD1). The higher preference for interaction of Phe CE2 atom is a result of the higher exposure of this atom to the solvent, and thus to an incoming bond. Conversely, the less exposed atom of Glu, namely OE1, is preferred over OE2, though we have no good explanation for this phenomenon. In the case of Leu, the atom notation is similar to Val. We have noticed that the CD1–CD1 distance is more occupied at 4 Å compared to CD2–CD2 distance (Figure 4E). It is more difficult to explain this situation, since both CD1 and CD2 are closer to the backbone oxygen than to the backbone nitrogen (data not shown). The mean distance values for the CD1, CD2 atoms to the backbone oxygen are 4.36 Å and 4.15 Å respectively, and to the backbone nitrogen 5.03 Å and 4.87 Å. Favoring the contact of CD1–CD1 might be a result of an unfavorable close contact between the two backbone oxygens. The last example we report is Pro, the atom notation of this residue is trivial. We have noticed different distance disruptions for two Pro carbon atoms CD and CB. In the distribution of Asn and Pro, the OD1–CD distance is more occupied compared to the OD1–CB distance at 3.5 Å (Figure 4F). The origin for this discrepancy might be the proximity of CD to the backbone nitrogen. Similar results were seen for Asp, Glu and Gln residues in contact with Pro.

### High-resolution X-ray structures provide very tight 4-D distributions

**Figure 5:**
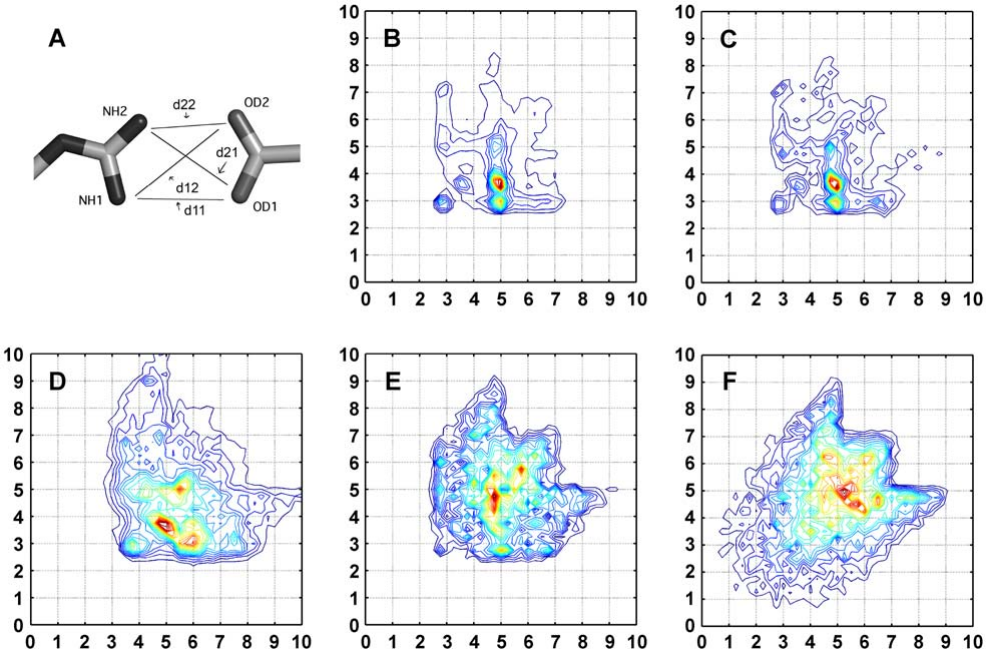
4-D histograms of the Arg–Asp pair (d12 *versus* d21) from different data sources. (A) The four distances for the Arg–Asp residue pair. (B) High-resolution X-ray data (<2 Å). (C) High-resolution X-ray data (<2 Å) collected at temperature between 275 to 300 Kelvin. (D) Low resolution X-ray data (2.5–3.0 Å). (E) NMR data. (F) Same data set as in (A) though all rotamers were randomly shuffled.

X-ray crystallography and NMR are the two main methods for protein structure determination at high resolution; in both, computational energy minimization is required at various stages of structure calculation. Since our distance potential was originated from high-resolution data, and the atom positions are highly restricted due to the 4-D data description of each residue pair, we argue that our new potential provides a realistic view of side chain orientations that can be used to evaluate computational minimization techniques. Figure 5 shows the NH1–OD1/NH2–OD2 distances for the Arg–Asp pair, based on distances collected from structures obtained from either high or low-resolution X-ray data, or from NMR. The high-resolution X-ray data gave the sharpest peaks, followed by the low-resolution data. Additionally we collected high-resolution X-ray data where the diffractions were collected at a temperature of 275–300 Kelvin. The 4-D data collected from NMR structures did not show any clear peaks, and resembled the distribution obtained from random data. It is argued that the proteins in NMR solutions are more flexible since the data is collected at room temperature. However, this should not affect the contact geometries, moreover, it seem that high resolution data collected at higher temperature do not change the distributions significantly (compare Figure 5B and 5C). What distinguishes high resolution X-ray data from low-resolution X-ray data and more so from NMR is the extent of minimization methods dictating the structure. This would suggest that the current minimization methods do not produce the “real” inter-residue contacts as provided by high-resolution X-ray structures. In the case of high-resolution structures the atom positions can be deduced more precisely from the electron density, which reduces the need for inaccurate minimization protocols [5].

### Generating a knowledge-based potential from the 4-D data histograms

Our initial aim in acquiring the 4-D distributions was to generate a knowledge-based potential. The standard KBP is built using Equation 1 and 2. Equation 1 is the conditional probability of a distance set in case where the amino acid sequence is known. The probability is defined as the product of the data collected (the probability of the four distances for a given amino acid pair) and the probability of an amino acid pair in the protein. Since the energy is estimated as the ratio of P_real_ and P_rand_ (Equation 2) and the real and random probabilities were generated from the same data set, the probability of the amino acid pair term cancels out. Equation 2 is an inverse Boltzmann relation [42], where Δ*E* is a pseudo energy gap obtained from the log ratio of the real and random distributions. As we do not attempt to predict experimental results, we defined *k*_B_T as unity. To calculate P_rand_ we used Equation 1 on the set of randomized structures as described in Methods. Our random model corresponds to the situation, in which conformations of side chains are not dictated by forces characteristic to real proteins. The random model would correspond to the starting point in the conformational search, in which residue side chain conformations are assigned arbitrarily. Thus this model is designed to maximize the difference between real interactions and the initial state of the system. According to Equation 2, three possible relations are found between the real and random distributions; in the case of a favorable interaction, P_real_ is higher than P_rand_, for an unfavorable interaction the opposite is observed. The third possibility is that both distributions are equal (neutral conformation) with a pseudo energy score of zero. The random distribution acts as a reference that distinguishes the preferred conformational states in the PDB database.
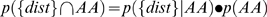
(1)

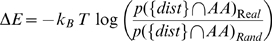
(2)


**Figure 6:**
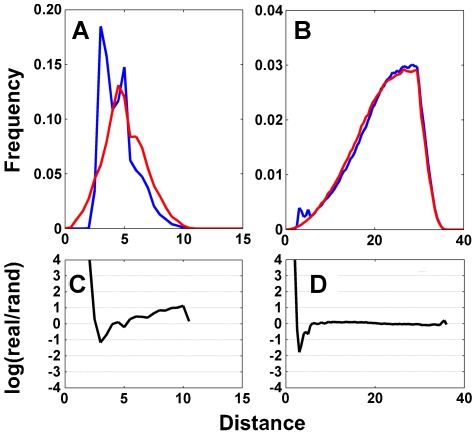
Building a random model for the KBP. (A) Data collected using a 5 Å cutoff (i.e. at least one distance <5 Å) for real data (blue) and random data (red). (B) Data collected using a 30 Å cutoff. (C) and (D) Log ratio of real and random distribution for (A) and (B) respectively.

A general phenomenon, which is not exclusive to the 4-D data, stems from the normalization of both the real and random distributions to one (or the same number of events are used for both sets). As bond distances in the real distribution peak at short distances (which reflect the bound state), while the random distribution of inter-residue distances will increase monotonically (Figure 6A), the curves of the two distributions have to cross each other at larger distances (as the integral of both curves equals to one). This causes positive pseudo energy (i.e. unfavorable conformation according to Equation 2) at larger distances, while no real interaction is expected (namely zero energy [13]). We observed the same unphysical pseudo energy gap values even when generating a random model accounting for both rotamer probabilities and atom clashes (data not shown). Because the number of counted interactions is growing with the square of the distance, even a small repulsive term at extended distances will have a large effect on the total energy calculated. We devised two solutions for this problem; the first was to raise the distance cutoff of the collected data to 30 Å, resulting in a much-reduced negative energy term (see Figure 6B versus 6A). This is better seen in Figures 6C and 6D, where the log ratio of the real/random is drawn, showing values much closer to zero for the raised cutoff. Secondly, we defined a distance cutoff for plausible interactions at 5 Å (at least one of the four distances has to be <5 Å for it to be counted). This distance cutoff removes all residue pairs with no direct interaction between them.

### Bin occupancy

**Figure 7:**
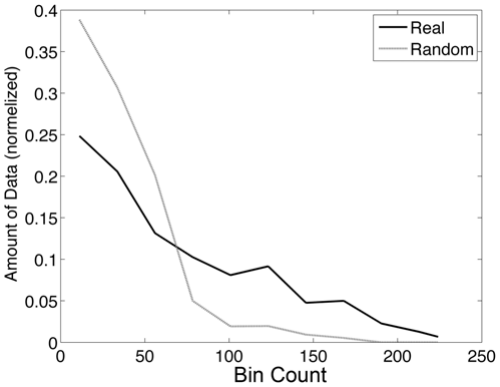
Bin count distribution. Bin counts for the real and random distributions of the Leu–Val pair. The y-axis is the normalized amount of data. The amount of data is the product of the number of measurements in a certain bin times the number of bins with the same number of measurements in this histogram.

Binning data at intervals of 0.5 Å gives a total number of 194481 bins up to a residue-residue distance of 10 Å. However, the number of “real” counts as extracted from the database was only between 1836 and 108094 for the least (Cys–Glu) and most frequent (Leu–Leu) amino acid pairs respectively, with a mean value of 108094 counts for all 190 interactions (see Table S1 for the entire dataset). Thus, most bins were actually empty, or were occupied by a very small number of events. This is demonstrated in Figure 7, where the amount of data is defined as the product of bin count times the number of bins with that count. For example, if N bins are occupied each by 5 events, the total number of events in this group is 5 N. To determine the distribution of events in different bins, the data was normalized by the total number of measurements in the histogram. The plot in Figure 7 shows the normalized data (y-axis) in bins with a particular number of counts (x-axis) for real and random distributions. Comparing the real and random bin occupancy distributions clearly shows that the random is dominated by low bin occupancies, while high counts are reserved for the real data. For low number of events per bin, the real and random data overlap. For example, the average count of events per bin for the Arg–Asp pair is 3.2±9, with some bins having up to 431 counts, whereas in the random data no bin has more than 19 counts. This suggests that the data generated from the bins with low occupancy are more prone to error (for example, if a bin of real data has 5 events, and the random has 2, the two occupancies are within the error of one another). The low number of average bin counts is a major issue of the 4-D method.

### Selecting preferred residue conformation

**Figure 8:**
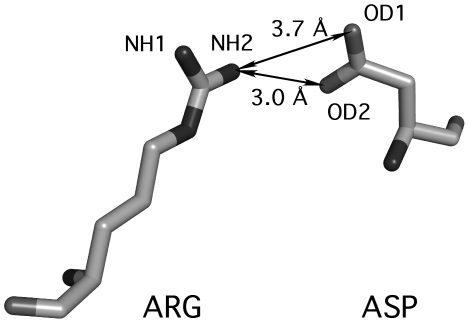
Optimal arrangement of Arg and Asp side chains simulated from the derived 4-D histograms. In the preferred orientation the carboxyl group of Asp is closer to NH2 of Arg than to NH1. To generate the depicted orientation, two residues in arbitrary conformation were placed randomly relative to each other, multiple times. The top scoring orientation, according to Equation 2, was identified and drawn in the figure. This simulation was repeated several times resulting in similar asymmetric placement of Arg and Asp side chains.

To evaluate whether the derived 4-D data can be used for modeling, a simple simulation was performed (see Methods for details). Figure 8 shows the 4-D histograms of an optimal side chain placement for Arg and Asp according to Equation 2. It is important to mention that the carboxyl group of Asp is always placed close to the NH2 atom of Arg. The same geometry for this contact is observed in high-resolution protein structures as was discussed above. The ability to reproduce the geometry of side chain/side chain contacts as observed in the PDB demonstrates that constructed 4-D histograms might be a useful tool for accurate protein structure modeling.

## Discussion

Knowledge-based potentials have become very popular and successful in recent years. For a KBP to be general, each term in it should have the maximal information content, and the number of terms should be minimized. Too many terms will make the energy landscape too ragged and cause overcounting. For example, a function that contains both a van der Waals term and an environment term may overcount the London dispersion force. We argue that the 4-D description introduced here has higher information content than the standard 1D distance usually used in KBPs, therefore providing a better definition for the relations (forces) between residues. The downside of a more exact, multi dimensional description of a structure is the ruggedness of the resulting energy surface, which cannot be exhaustively probed using discrete rotamers. Moreover, to properly define all the interactions requires an amount of data currently not available. Therefore, implementing the four-distance description for sampling requires reducing the potential ruggedness, which can be achieved by smoothening the data (Potapov, V., Cohen, M. & Schreiber, G.; unpublished data, 2009). However, for scoring, the ruggedness is of lesser importance, thus the original histograms can be used.

The high information content of amino acid interactions was demonstrated by the observation that many such interactions are asymmetric, a surprising fact by itself. The asymmetry involved interactions of Arg, Glu, Val, Phe, His, Leu, Pro and Ile. To verify that the asymmetry is not a result of inconsistent naming we verified that the atom names were assigned according to the IUPAC definitions [65]. For seven residues, in which branches are identical, we found that substantial amount of atom name assignments in the PDB do not follow the conventions; 24% of the cases for Phe, 23% for Tyr, 18% for Glu, 15% for Asp, 8% for Arg, 0.8% for Leu, and 0.01% for Val. We could not attribute this to particular structural refinement software or the date of deposition.

This kind of asymmetry was not detected previously, using 1D data or physical forcefields. However, the asymmetry was also not detected in KBP, which use information on bond lengths, bond angles, and dihedral angles for pairs, triplets, and quandruplets of bonded atoms. The reason for this may be the lower information content of other KBPs. A recent forcefield developed by Ma *et al.* [59] is actually a 3D potential where the geometry between the volume blocks is defined by two angles and a distance between two planes. This method also cannot detect the asymmetry of the residue atoms since it is a type of united atom representation. For example, NH1 and NH2 belong to the same volume block. The reasoning behind the asymmetry given here is mostly intuitive. We do not argue that there is a chemical difference between different residue atoms. Though we argue that a KBP that is based on the PDB must maximize the information content extracted from it, thus, this asymmetry must be taken into account. The advantage of KBP over physical based potentials is the fact that unknown factors can emerge from the constructed potentials, with no actual known physical explanation. One example for such is the hydrogen bond geometry reported by Kortemme *et al.* [66]. The physical model predicted the angle between the hydrogen acceptor and the atom covalently bound to the acceptor to be 180°, while in the PDB most of these angles are closer to 120°. A KBP taking advantage of the full extent of information in the PDB database may be better in modeling protein structures. More exact calculations in the future will be needed to produce a more satisfying explanation for the observed asymmetries. However, the advantage of KBPs over physical forcefields is that one can use observations to model protein structures, even if they are not fully understood.

Since most side chains contain more than two atoms, we had to decide which of the atoms to use to generate the 4-D data. For example the structurally simple Val-Val pair has three different atom pairs CB–CG1, CB–CG2 and CG1–CG2. Thus, 6 different 4 atoms distances could be generated. More generally, the 202 different atom pairs on the 19 amino acids (excluding Gly) can be paired using 20503 different 4-atom distributions. To choose the 190 representative pairs used for the 4-D database (one per residue pair) we considered three different approaches; first, manually choosing the pair that best represents the residue (e.g. CG1–CG2 for Val or OD1–ND2 of Asn). The second approach was to maximize the Kullback–Leibler divergence between the real and the corresponding random distributions. Since the Kullback–Leibler divergence measures the difference between two probabilities the pair with the highest difference may have the highest information content. The third approach was to search for the pair with the highest number of accepted distance measurements (at least one distance less than 5 Å). The problem of limited data is clearly demonstrated in Figure 6, that shows that most bins are empty, or contain only low bin counts that are statistically within the numbers found when using random side chain rotamers. This is a main problem with a KBP, as the division between two small numbers (random and real bin occupancy) still generates a value different from 1, and thus assigns an energy term to that bin. To minimize this problem, we currently use the most frequent atom pairs as described above. Using this logic to choose atom pairs also seems to produce the best results when using the 4-D matrix for side chain modeling (Potapov, V., Cohen, M. & Schreiber, G.; unpublished data, 2009). This may be the result of the limited amount of data in the PDB, and thus may change once many more structures will be available.

## Supporting Information

Table S1 Supporting Table
(0.38 MB DOC)
