# Supplementary material for: Four Distances between Pairs of Amino Acids Provide a Precise Description of their Interaction
Source: PLoS Comput Biol. 2009 Aug 14;5(8):e1000470. doi: 10.1371/journal.pcbi.1000470 (PMC2715887; doi:10.1371/journal.pcbi.1000470)
Supplement: Table S1 — Supporting Table (0.38 MB DOC) [file pcbi.1000470.s001.doc]

Table S1. The amount of occurrences of all pairwise interactions in the database.

| R1 | R2 | A1 | A2 | B1 | B2 | Number of measurements |
| --- | --- | --- | --- | --- | --- | --- |
| **A. 190 side chain-side chain interactions** | | | | | | |
| ALA | ALA | CB | CB | CB | CB | 11963 |
| ALA | ARG | CB | CB | CB | CD | 7442 |
| ALA | ASN | CB | CB | CB | ND2 | 8475 |
| ALA | ASP | CB | CB | CB | OD1 | 10666 |
| ALA | CYS | CB | CB | CB | SG | 4842 |
| ALA | GLN | CB | CB | OE1 | NE2 | 7160 |
| ALA | GLU | CB | CB | CB | OE2 | 9430 |
| ALA | HIS | CB | CB | ND1 | NE2 | 5303 |
| ALA | ILE | CB | CB | CG2 | CD1 | 29843 |
| ALA | LEU | CB | CB | CD1 | CD2 | 48254 |
| ALA | LYS | CB | CB | CB | CE | 6879 |
| ALA | MET | CB | CB | CG | CE | 8212 |
| ALA | PHE | CB | CB | CE1 | CE2 | 19240 |
| ALA | PRO | CB | CB | CB | CD | 10634 |
| ALA | SER | CB | CB | CB | OG | 13263 |
| ALA | THR | CB | CB | CG2 | OG1 | 15673 |
| ALA | TRP | CB | CB | CB | CH2 | 6259 |
| ALA | TYR | CB | CB | CE1 | OH | 10960 |
| ALA | VAL | CB | CB | CG2 | CG1 | 34268 |
| ARG | ARG | CG | NH2 | CD | NH2 | 9787 |
| ARG | ASN | CG | NH2 | OD1 | ND2 | 8829 |
| ARG | CYS | CG | NH2 | CB | SG | 2360 |
| ARG | GLN | CG | NH2 | OE1 | NE2 | 8081 |
| ARG | GLU | CD | NH2 | OE1 | OE2 | 26838 |
| ARG | HIS | CG | NH2 | ND1 | NE2 | 4817 |
| ARG | ILE | CG | NH2 | CG2 | CD1 | 11568 |
| ARG | LEU | CG | NH2 | CD1 | CD2 | 20795 |
| ARG | LYS | NH1 | NH2 | CB | NZ | 5903 |
| ARG | MET | CG | NH2 | SD | CE | 4005 |
| ARG | PHE | CG | NH2 | CE1 | CE2 | 9129 |
| ARG | PRO | NH1 | NH2 | CB | CD | 7930 |
| ARG | SER | CG | NH2 | CB | OG | 10231 |
| ARG | THR | CG | NH2 | CG2 | OG1 | 10731 |
| ARG | TRP | CG | NH2 | CD1 | CH2 | 4791 |
| ARG | TYR | CG | NH2 | CE1 | OH | 10410 |
| ARG | VAL | CG | NH2 | CG2 | CG1 | 13429 |
| ASN | ASN | CB | OD1 | OD1 | ND2 | 10366 |
| ASN | ASP | OD1 | ND2 | OD1 | OD2 | 12087 |
| ASN | CYS | CB | OD1 | CB | SG | 2187 |
| ASN | GLN | OD1 | ND2 | OE1 | NE2 | 6838 |
| ASN | GLU | OD1 | ND2 | OE1 | OE2 | 9287 |
| ASN | HIS | OD1 | ND2 | ND1 | NE2 | 4080 |
| ASN | ILE | OD1 | ND2 | CG2 | CD1 | 8190 |
| ASN | LEU | CB | ND2 | CD1 | CD2 | 11784 |
| ASN | LYS | OD1 | ND2 | CG | NZ | 8943 |
| ASN | MET | CB | ND2 | CB | CE | 3053 |
| ASN | PHE | OD1 | ND2 | CG | CZ | 6333 |
| ASN | PRO | OD1 | ND2 | CB | CD | 6279 |
| ASN | SER | OD1 | ND2 | CB | OG | 10250 |
| ASN | THR | OD1 | ND2 | CG2 | OG1 | 10664 |
| ASN | TRP | CB | ND2 | NE1 | CZ3 | 3311 |
| ASN | TYR | OD1 | ND2 | CE1 | OH | 6701 |
| ASN | VAL | CB | ND2 | CG2 | CG1 | 9375 |
| ASP | ASP | CB | OD2 | OD1 | OD2 | 10370 |
| ASP | CYS | CB | OD1 | CB | SG | 2308 |
| ASP | GLN | OD1 | OD2 | OE1 | NE2 | 7779 |
| ASP | GLU | OD1 | OD2 | OE1 | OE2 | 7920 |
| ASP | HIS | OD1 | OD2 | ND1 | NE2 | 7799 |
| ASP | ILE | CB | OD2 | CG2 | CD1 | 8600 |
| ASP | LEU | CB | OD2 | CD1 | CD2 | 13231 |
| ASP | LYS | OD1 | OD2 | CG | NZ | 21605 |
| ASP | MET | CB | OD2 | CG | CE | 3109 |
| ASP | PHE | CB | OD1 | CE1 | CE2 | 6204 |
| ASP | PRO | CB | OD1 | CB | CD | 7074 |
| ASP | SER | OD1 | OD2 | CB | OG | 16006 |
| ASP | THR | OD1 | OD2 | CG2 | OG1 | 14539 |
| ASP | TRP | CB | OD1 | NE1 | CH2 | 3431 |
| ASP | TYR | OD1 | OD2 | CE1 | OH | 9620 |
| ASP | VAL | CB | OD2 | CG2 | CG1 | 10651 |
| CYS | CYS | CB | SG | CB | SG | 5706 |
| CYS | GLN | CB | SG | CB | NE2 | 1902 |
| CYS | GLU | CB | SG | CG | OE1 | 1901 |
| CYS | HIS | CB | SG | ND1 | NE2 | 1836 |
| CYS | ILE | CB | SG | CG2 | CD1 | 6405 |
| CYS | LEU | CB | SG | CD1 | CD2 | 10197 |
| CYS | LYS | CB | SG | CB | CE | 1903 |
| CYS | MET | CB | SG | CG | CE | 2214 |
| CYS | PHE | CB | SG | CE1 | CE2 | 4758 |
| CYS | PRO | CB | SG | CB | CD | 3253 |
| CYS | SER | CB | SG | CB | OG | 3390 |
| CYS | THR | CB | SG | CG2 | OG1 | 3445 |
| CYS | TRP | CB | SG | CB | CH2 | 2012 |
| CYS | TYR | CB | SG | CE1 | OH | 2360 |
| CYS | VAL | CB | SG | CG2 | CG1 | 7328 |
| GLN | GLN | CG | OE1 | OE1 | NE2 | 5546 |
| GLN | GLU | OE1 | NE2 | OE1 | OE2 | 7411 |
| GLN | HIS | OE1 | NE2 | ND1 | NE2 | 3323 |
| GLN | ILE | CB | NE2 | CG2 | CD1 | 8266 |
| GLN | LEU | CB | NE2 | CD1 | CD2 | 14241 |
| GLN | LYS | OE1 | NE2 | CG | NZ | 7096 |
| GLN | MET | CB | NE2 | CG | CE | 2926 |
| GLN | PHE | CB | NE2 | CE1 | CE2 | 6269 |
| GLN | PRO | OE1 | NE2 | CB | CD | 5891 |
| GLN | SER | OE1 | NE2 | CB | OG | 7558 |
| GLN | THR | OE1 | NE2 | CG2 | OG1 | 8323 |
| GLN | TRP | CB | NE2 | NE1 | CH2 | 3338 |
| GLN | TYR | OE1 | NE2 | CE1 | OH | 5848 |
| GLN | VAL | CB | NE2 | CG2 | CG1 | 9208 |
| GLU | GLU | CG | OE2 | OE1 | OE2 | 8219 |
| GLU | HIS | OE1 | OE2 | ND1 | NE2 | 7400 |
| GLU | ILE | CB | OE2 | CG2 | CD1 | 11176 |
| GLU | LEU | CG | OE1 | CD1 | CD2 | 17857 |
| GLU | LYS | OE1 | OE2 | CD | NZ | 23818 |
| GLU | MET | CG | OE1 | CG | CE | 3567 |
| GLU | PHE | CB | OE2 | CE1 | CE2 | 8043 |
| GLU | PRO | OE1 | OE2 | CB | CG | 7699 |
| GLU | SER | OE1 | OE2 | CB | OG | 12362 |
| GLU | THR | OE1 | OE2 | CG2 | OG1 | 12551 |
| GLU | TRP | CG | OE1 | NE1 | CH2 | 4215 |
| GLU | TYR | OE1 | OE2 | CE1 | OH | 9939 |
| GLU | VAL | CB | OE2 | CG2 | CG1 | 12426 |
| HIS | HIS | ND1 | NE2 | ND1 | NE2 | 2564 |
| HIS | ILE | ND1 | NE2 | CG2 | CD1 | 5543 |
| HIS | LEU | ND1 | NE2 | CD1 | CD2 | 9601 |
| HIS | LYS | ND1 | NE2 | CG | NZ | 3072 |
| HIS | MET | ND1 | NE2 | SD | CE | 2299 |
| HIS | PHE | ND1 | NE2 | CE1 | CE2 | 4898 |
| HIS | PRO | ND1 | NE2 | CB | CD | 4767 |
| HIS | SER | ND1 | NE2 | CB | OG | 5715 |
| HIS | THR | ND1 | NE2 | CG2 | OG1 | 5628 |
| HIS | TRP | ND1 | NE2 | CD1 | CH2 | 2499 |
| HIS | TYR | ND1 | NE2 | CE1 | OH | 5465 |
| HIS | VAL | ND1 | NE2 | CG2 | CG1 | 6561 |
| ILE | ILE | CB | CD1 | CG2 | CD1 | 40455 |
| ILE | LEU | CG2 | CD1 | CD1 | CD2 | 75263 |
| ILE | LYS | CG2 | CD1 | CB | CE | 10111 |
| ILE | MET | CG2 | CD1 | SD | CE | 11829 |
| ILE | PHE | CG2 | CD1 | CE1 | CE2 | 28231 |
| ILE | PRO | CG2 | CD1 | CB | CD | 12178 |
| ILE | SER | CG2 | CD1 | CB | OG | 12408 |
| ILE | THR | CG2 | CD1 | CG2 | OG1 | 17743 |
| ILE | TRP | CG2 | CD1 | CB | CH2 | 8753 |
| ILE | TYR | CG2 | CD1 | CE1 | OH | 13392 |
| ILE | VAL | CG2 | CD1 | CG2 | CG1 | 45638 |
| LEU | LEU | CG | CD1 | CD1 | CD2 | 108094 |
| LEU | LYS | CD1 | CD2 | CB | CE | 15587 |
| LEU | MET | CD1 | CD2 | SD | CE | 19570 |
| LEU | PHE | CD1 | CD2 | CE1 | CE2 | 48883 |
| LEU | PRO | CD1 | CD2 | CB | CD | 19836 |
| LEU | SER | CD1 | CD2 | CB | OG | 18304 |
| LEU | THR | CD1 | CD2 | CG2 | OG1 | 26679 |
| LEU | TRP | CD1 | CD2 | CB | CH2 | 15438 |
| LEU | TYR | CD1 | CD2 | CE1 | OH | 22786 |
| LEU | VAL | CD1 | CD2 | CG2 | CG1 | 78298 |
| LYS | LYS | CB | NZ | CG | NZ | 4326 |
| LYS | MET | CB | CE | SD | CE | 3150 |
| LYS | PHE | CB | CE | CE1 | CE2 | 7625 |
| LYS | PRO | CB | NZ | CB | CD | 4830 |
| LYS | SER | CB | NZ | CB | OG | 9341 |
| LYS | THR | CG | NZ | CG2 | OG1 | 8975 |
| LYS | TRP | CB | CE | CD1 | CH2 | 3447 |
| LYS | TYR | CG | NZ | CE1 | OH | 8952 |
| LYS | VAL | CB | CE | CG2 | CG1 | 10911 |
| MET | MET | CG | CE | SD | CE | 4772 |
| MET | PHE | SD | CE | CE1 | CE2 | 9241 |
| MET | PRO | SD | CE | CB | CD | 4335 |
| MET | SER | CG | CE | CB | OG | 4022 |
| MET | THR | CG | CE | CG2 | OG1 | 5497 |
| MET | TRP | CG | CE | CB | CH2 | 3309 |
| MET | TYR | CG | CE | CE1 | OH | 5070 |
| MET | VAL | CG | CE | CG2 | CG1 | 12356 |
| PHE | PHE | CE1 | CE2 | CG | CZ | 22476 |
| PHE | PRO | CE1 | CE2 | CB | CD | 11027 |
| PHE | SER | CE1 | CE2 | CB | OG | 9306 |
| PHE | THR | CE1 | CE2 | CG2 | OG1 | 11247 |
| PHE | TRP | CE1 | CE2 | CB | CH2 | 7656 |
| PHE | TYR | CE1 | CE2 | CE1 | OH | 11059 |
| PHE | VAL | CE1 | CE2 | CG2 | CG1 | 28611 |
| PRO | PRO | CB | CG | CB | CD | 7038 |
| PRO | SER | CB | CD | CB | OG | 8703 |
| PRO | THR | CB | CD | CG2 | OG1 | 9713 |
| PRO | TRP | CB | CD | CD1 | CH2 | 6178 |
| PRO | TYR | CB | CD | CE1 | OH | 11393 |
| PRO | VAL | CB | CD | CG2 | CG1 | 15216 |
| SER | SER | CB | OG | CB | OG | 7179 |
| SER | THR | CB | OG | CG2 | OG1 | 13601 |
| SER | TRP | CB | OG | CD1 | CH2 | 4230 |
| SER | TYR | CB | OG | CE1 | OH | 7701 |
| SER | VAL | CB | OG | CG2 | CG1 | 14887 |
| THR | THR | CB | CG2 | CG2 | OG1 | 12848 |
| THR | TRP | CG2 | OG1 | CD1 | CH2 | 4475 |
| THR | TYR | CG2 | OG1 | CE1 | OH | 8349 |
| THR | VAL | CG2 | OG1 | CG2 | CG1 | 20152 |
| TRP | TRP | CB | CH2 | CD1 | CH2 | 3273 |
| TRP | TYR | CD1 | CH2 | CE1 | OH | 4226 |
| TRP | VAL | CB | CH2 | CG2 | CG1 | 9365 |
| TYR | TYR | CE1 | OH | CE1 | OH | 3615 |
| TYR | VAL | CE1 | OH | CG2 | CG1 | 14145 |
| VAL | VAL | CB | CG2 | CG2 | CG1 | 41167 |
| **B. 18 side chain-main chain interactions** | | | | | | |
| ARG | ANY | CG | NH2 | CA | O | 192608 |
| ASN | ANY | OD1 | ND2 | CA | O | 149885 |
| ASP | ANY | OD1 | OD2 | CA | O | 170024 |
| CYS | ANY | CB | SG | CA | O | 68565 |
| GLN | ANY | OE1 | NE2 | CA | O | 114873 |
| GLU | ANY | CB | OE2 | CA | O | 171096 |
| HIS | ANY | ND1 | NE2 | CA | O | 75826 |
| ILE | ANY | CG2 | CD1 | CA | O | 238788 |
| LEU | ANY | CD1 | CD2 | CA | O | 365429 |
| LYS | ANY | CB | NZ | CA | O | 178102 |
| MET | ANY | CB | CE | CA | O | 84484 |
| PHE | ANY | CE1 | CE2 | CA | O | 158945 |
| PRO | ANY | CB | CD | CA | O | 179277 |
| SER | ANY | CB | OG | CA | O | 204785 |
| THR | ANY | CG2 | OG1 | CA | O | 210590 |
| TRP | ANY | NE1 | CZ3 | CA | O | 70789 |
| TYR | ANY | CE1 | CE2 | CA | O | 130920 |
| VAL | ANY | CG2 | CG1 | CA | O | 283691 |

R1 – First residue

R2 – Second residue

A1 – First atom from R1

A2 – Second atom from R1

B1 – First atom from R2

B2 – Second atom from R2
